# The role of the agricultural sector in Ghanaian development: a multiregional SAM-based analysis

**DOI:** 10.1186/s40008-022-00265-9

**Published:** 2022-06-20

**Authors:** Valeria Ferreira, Miguel Ángel Almazán-Gómez, Victor Nechifor, Emanuele Ferrari

**Affiliations:** 1 Department of Business Management, Universitat Rovira I Virgili, Reus, Spain; 2 Department of Economic Analysis, University of Zaragoza, Saragossa, Spain; 3 European Commission, Joint Research Centre (JRC), Seville, Spain

**Keywords:** Social Accounting Matrix (SAM), Multiplier analysis, Multiregional, Structural path analysis, Agriculture, Ghana, Inequality

## Abstract

Ghana shows remarkable differences in employment and welfare between the southern and northern regions. The promotion of policy focus on the development of the northern regions requires the elaboration of specific databases describing the regional economies. Hence, this work outlines the construction of a Social Accounting Matrix (SAM) for Ghana for the year 2015 with a high disaggregation of sectors, household income groups and education levels across 10 administrative regions. Linear multisectoral models have been applied to this SAM to estimate socio-economic impacts of potential final demand policies down to a regional level in the Ghanaian economy. Further on, the structural path analysis is used to investigate the role played by different agriculture commodities in transmitting income to different types of households. The results allow for an identification of the most suitable sectors to be promoted due to their ability to generate the highest increases in output, employment and value added in the rest of the economy, as well as those with a significant impact on household income generation. As a result, the primary sector will play a key role in the economic and employment growth of the country. Notably, sorghum and millet, pulses, tobacco, cotton and fibres can be considered favourable crops for development in the Northern region.

## Introduction

The Ghanaian economy is experiencing a structural change with an increasing importance of the services sector in the GDP. However, Ghana’s primary sector accounts for 19% of the GDP and employs 34% of the workforce. It is also an important source of income for many households (GSS 2017) so it remains essential to the country's sustainable growth and development because of its impact on employment, income generation and poverty reduction (Mensah 2019; Danso-Abbeam and Baiyegunhi 2020). Maize, root, vegetables and fruits, cassava, cocoa and forestry are the main primary activities. Ghana is the second most important producer and exporter of cocoa in the world after Ivory Coast. Cocoa is very important in terms of foreign exchange, employment, and a key driver of economic growth (Danso-Abbeam and Baiyegunhi 2020).

In Ghana, as in other developing countries, households play a dual role as consumers as well as producers of commodities. As in a typical subsistence agriculture sector, households produce for their own consumption and for the market. Based on the Ghana Living Standards Survey (GLSS), Ghanaian households show notable differences in employment and welfare across regions, with the northern regions^1^ having the lowest per capita incomes.

As part of Ghana’s Strategy Support Programme Growth, the Poverty Reduction Strategy, Medium-Term Agriculture Sector Investment Plan (METASIP) and the Food and Agriculture Sector Development Policy (FASDEP), Ghana designed policies to develop the agricultural, fishing, and forestry sectors with a main focus on the development of the Northern region (World Bank 2017). Agricultural development has positive effects on food security, the supply of inputs for the food industry, the creation of employment and the generation of foreign exchange earnings (Danso-Abbeam et al. 2019).

According to the Ghana Strategy Support Programme (GSSP), promoting agricultural growth will have a great effect on reducing poverty at the regional level because of the strong linkages between income and consumption. Stimulating agricultural output would lead the growth and the structural transformation of the Ghanaian economy (IFPRI 2005). To this end, actions should focus on promoting suitable commodities to drive economic growth, employment, and income diversification, while taking into account the reduction of regional inequalities.

To promote the expected development, these policies must be based on knowledge and on the analysis of the sectors and agents of the economy and the linkages between them. The analysis of the Ghanaian economy requires a specific database that describes the regional economies, with the primary sector disaggregated, and considers the interlinkages between all agents and regions. This paper employs a Social Accounting Matrices (SAM) for Ghana (Ferreira et al. 2021) which is highly disaggregated across sectors, regions, income sources and education levels and which accounts for ‘home production for home consumption’ (HPHC) commodities.

A SAM is a database that shows the interaction between production, income, consumption, and investment. The richness of the information in the SAM allows for an analysis of the linkages among sectors and all other agents and accounts of an economy. A SAM can be used to apply a multiplier analysis linking changes in demand in the economy to changes in output, factor incomes, household incomes and employment (Arndt et al. 2000).

Input–output tables have been used to calculate multipliers for the Ghanaian economy (Nchor 2014; Bentum-Ennin 2018) and to analyse the sectoral linkages for the specific case of the oil sector (Nchor and Konderla 2016). However, due to the characteristics of the Ghanaian economy, the SAM multiplier models are more appropriated tools, as they allow closing the circular flow of income and expenditure, taking into account household-related socio-economic data from household budget and labour force surveys. The first SAM for Ghana, published for 1993 (Powell and Round 1998)**,** has been used to analyse redistribution and poverty (Bussolo and Round, 2003; Arbenser 2004)**.** The SAMs for 1999 and for 2013 were constructed and used to analyse the impact of trade liberalisation on income distribution in Ghana using CGE models (Bhasin and Obeng 2007; Mensah 2019). More recently, a 2015 SAM with a standard structure was used to estimate the economic impact of COVID-19 in Ghana (Amewu et al. 2020) and to analyse two hypotheses related to the fertiliser subsidy programme (Iddrisu et al. 2019). None of the matrices have the regional breakdown, among other differences of the SAM used in this article.

In this regard, this article describes the SAM for Ghana 2015 that accounts for a high disaggregation with the regional peculiarities, suitable to provide policy-relevant analysis (Ferreira et al. 2021). The objective of this study is to analyse the impact of demand-side policies on the economy’ production, households’ income and job creation across the different regions of the country. More specifically, the study presents an analysis of multipliers effects using the 2015 SAM for Ghana and it completes the analysis by applying structural path and value chain analysis.

The analysis of the multipliers shows how much output will be generated in each activity of the economy due to exogenous impacts of new final demand, how many jobs will be created and the effect of the shock on household income. The structural path analysis provides a detailed description of the transmission mechanisms of the effects described for each multiplier. The results indicate those commodities with a greater potential to generate output, value added and employment. It also identifies the impact on the different regions of the country. This allows for the classification of commodities according to their capacity to generate economic growth, highlighting those regions and commodities to be tackled by policies focused not only on the country's development, but also specifically on equality and development in the neediest regions.

Cotton and fibres, sugar cane, sorghum and millet, maize, livestock, tobacco, roots, pulses, and groundnuts are the most relevant commodities with higher multiplier values in terms of value added, employment, output, and income. Moreover, sorghum and millet, pulses, tobacco, cotton and fibres, and livestock have more influence in the northern regions and are suitable for policy promotion for the development of the north of the country.

The rest of the paper is structured as follows. Section 2 introduces the HPHC approach, a key aspect of the new SAM employed, followed by a summary description of the structure disaggregation details of the Ghana SAM. Section 3 describes linear SAM models, explaining each multiplier with the structural path and value chain analysis to show the usefulness of SAMs in policy impact assessment. The results in Sect. 4 show the multiplier effects to assess the impact of final demand shocks on the economy in terms of output, value added, employment and household income. Section 5 presents the discussions and Sect. 6 concludes including policy recommendations.

## A highly disaggregated SAM for Ghana

A SAM is a comprehensive, economy-wide database to represent all the economic transactions carried out among the agents of a specific economy over a period, generally 1 year. It is thus a tool to understand the structure of an economy and a suitable database for economic modelling allowing the calculation of multipliers or the application of CGE models (Round 2003a; Burfisher 2016).

This study employs a highly disaggregated SAM for Ghana (base year 2015) (Ferreira et al. 2021). The SAM includes specific accounts for the treatment of HPHC, a high disaggregation based on the country regions, among other peculiarities. This framework allows addressing specific issues related to production and productive factors for each region distinguishing between north and south, and the interrelationship between the production structure and the distribution of incomes of different household groups, to reduce inequality within regions.

### Home production for home consumption (HPHC) approach and regional disaggregation

As the dual role of households as producers and consumers is a typical characteristic in Ghana, the SAM includes the HPHC approach by assuming that each household also acts as a commodities producing unit. That implies to separate the use of inputs for the production of these households as activities and the commodities produced by these households for own consumption and for the market (Aragie and McDonald 2014).

The agricultural production on the Savannah agro-ecological zone that includes mainly the regions in the north (Abbam et al. 2018) is constrained due to inadequate infrastructure, poor access to finance, among others socio-economic and climate conditions problems (World Bank 2017). As a result, the northern regions have a low per capita income and remain the least developed in the country. In this regard, the government promotes the agricultural potential of the northern regions to reduce inequality among the regions.

**Fig. 1:**
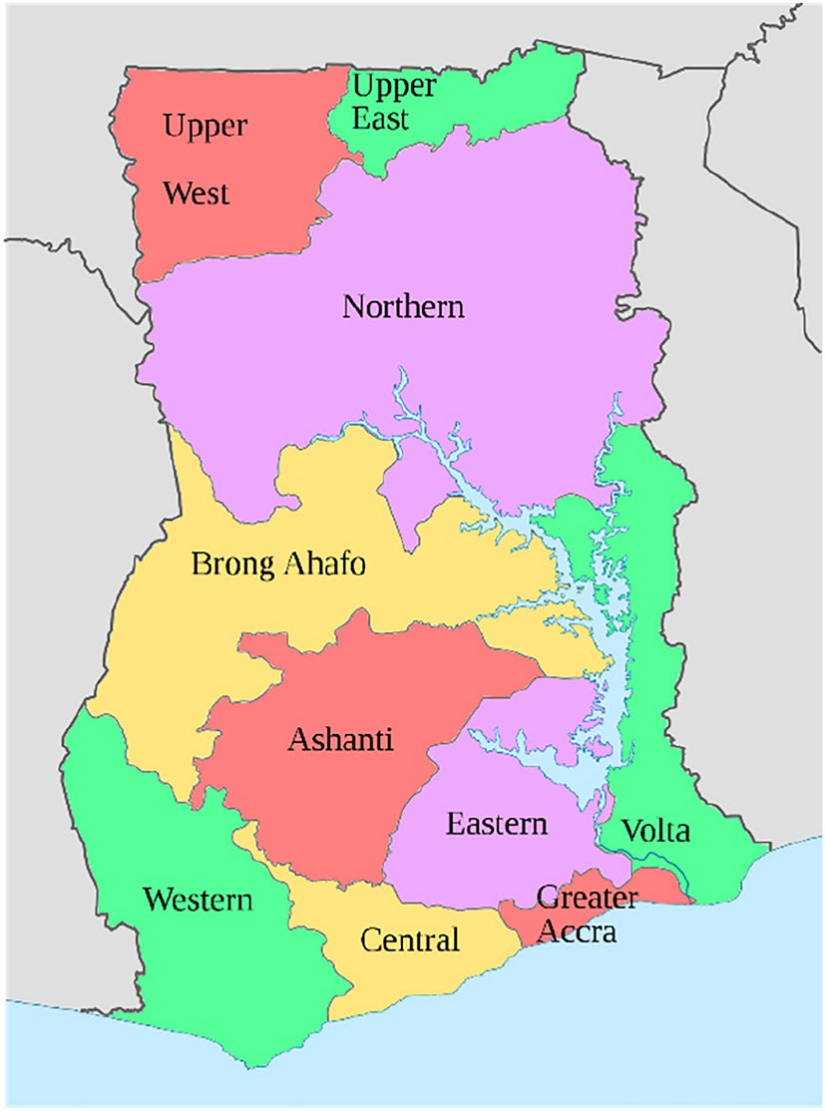
Administrative regions of Ghana (Source: http://wikimedia.org. File: Regions_of_Ghana_en.svg)

For this reason, one of the most important contribution of this SAM is the regional disaggregation into 10 regions (reflective of the sub-national boundaries prior to the 2018 referendum) that has been applied to both households, as productive units (activities), and as institutional units, and for labour and land factors. The regional breakdown in the 2015 Ghana SAM includes for the north of the country the Northern, Upper East and Upper West administrative regions. The south is defined by the Brong-Ahafo, Volta, Ashanti, Western, Central, Eastern and Greater Accra regions (GSS 2017) (see Fig. 1).

### Structure details

The SAM for Ghana is characterised by a high disaggregation carried out using the microdata provided by the 2016/2017 Ghana Living Standard Survey Round 7 (GLSS7) (GSS 2019) and the 2015 Labour Force Survey (GSS 2016)—see Ferreira et al. (2021) for more details.

The SAM accounts for an agricultural households’ activity for each region that produces 14 “subsistence commodities” and 20 marketed ones. The remaining 35 marketed commodities are produced by specific national activities. Household are disaggregated so that each region accounts for rural and urban representative household groups disaggregated by per capita expenditure quintiles. Such classification allows for an analysis of redistributive aspects and the specific impact of different policies in different regions. The factors of production are separated into three broad categories: labour, land and capital. The labour factor is disaggregated into three types of labour: skilled, semi-skilled and unskilled labour (based on educational attainment), regionalised in the 10 regions and further separated into rural and urban. Four types of capital are considered: crops, livestock, mining and other. Each region also accounts for a land factor (employed by agricultural crops). In summary, the 2015 Ghana SAM contains 299 accounts detail in Table 1. Moreover, the structure and a short version of the SAM is summarised in Table 2.

**Table 1:** Ghana SAM 2015 accounts. Source: own elaboration based on Ferreira et al. (2021)

Group	Detail	Accounts
Activities	Activities	10
	Households as activities	35
Commodities	Marketed commodities	14
	HPHC commodities	55
Margins	Trade and transport margins	2
Factors of production	Labour factors	60
	Capital factor	10
	Land factors	4
Private and public agents	Households	100
	Government	1
Taxes	Taxes—direct	1
	Taxes—export	1
	Taxes—import	1
	Taxes—sales	1
I-S	Savings–investment/change in stocks	2
ROW	Rest of the world	1
Total		299

**Table 2:** Ghana SAM 2015 with aggregated values, millions of Ghanaian Cedis. Source: Ferreira et al. (2021)

	CH	C	MARG	AH	A	LAB	LAN	CAP	HH	ENT	GOV	TAX	I-S	ROW	Total
CH				16					4547						4562
C			22,439	4213	123,349				84,099		22,341		38,504	52,257	347,203
MARG		22,439													22,439
AH	4562	21,662													26,225
A		223,194													223,194
LAB				8235	34,960										43,196
LAN				8387											8387
CAP				5373	64,885									781	71,038
HH						43,196	8387	4177		59,290	1571			4991	121,612
ENT								63,966			9020				72,986
GOV									290	6088		22,455		2775	31,607
TAX		15,063							3572	3819					22,455
I-S									29,104	3789	− 3053		2540	8665	41,044
ROW		64,844						2895			1729				69,468
Total	4562	347,203	22,439	26,225	223,194	43,196	8387	71,038	121,612	72,986	31,607	22,455	41,044	69,468	

*CH* HPHC commodities, *C* marketed commodities, *MARG* trade margins, *AH* HPHC activities, *A* market activities, *LAB* labour, *LAN* land, *CAP* capital, *HH* households, *ENT* enterprises, *GOV* government, *TAX* taxes, *I-S* investment–savings, *ROW* rest of the world

## Multipliers, value chain and structural path analysis

The richness of the information in the SAM allows for the analysis of the linkages among sectors and all other accounts. SAM models are simple tools to analyse the structure of an economy. They enable to evaluate the transmission mechanisms of potential shocks through economic sectors, showing the effects generated on the economic activity of different agents due to the relationships of the circular flow of income (Pyatt 1988).

To obtain a multiplier matrix that traces the impacts on the endogenous account of impulses on the exogenous accounts, it is necessary to define the endogenous and exogenous accounts of the model (Acharya 2007). Following their conventional classification, the exogenous accounts are the public sector, rest of the world and investments and savings, (Defourny and Thorbecke 1984; Pyatt and Round 1985). The standard representation of the multipliers matrix is as follows: ${\varvec{M}}={\left({\varvec{I}}-{\varvec{A}}\right)}^{-1}$, where the matrix ${\varvec{A}}$ is the coefficient matrix (calculating dividing each element of the endogenous accounts in the SAM by the total of their corresponding column); each element ${m}_{ij}$ in ${\varvec{M}}$ shows the requirements of account $i$ to increase the final demand of account $j$ by one monetary unit (Mainar-Causapé et al. 2020).

The analysis of the multipliers ranks commodities according to their capacity to generate economic growth and identifies those sectors that should receive more attention from policies. Multipliers allow knowing how much output will be generated in the economy and in each activity due to exogenous shocks, how many jobs will be created and the effect of the shock on households’ income (DiPasquale and Polenske 1980).

This paper focuses on the analysis of the economic impacts of new final demand in each commodity and its distributed effects among the different regions, measured by the variation on the output of the activities, the value added generated in the economy, the jobs created and the increase on households’ income (Miller and Blair 2009). To analyse these impacts, the output, employment, household income and value-added multipliers will be calculated.

The value chain analysis is a complementary step to provide information about the distribution of the value added generated to the activities of the economy due to an exogenous shock in a specific commodity demand. This analysis identifies which commodities that can be influenced with demand shocks will have more impact on specifics activities (Mainar-Causapé et al. 2020). Subsequently, a structural path analysis (SPA) is conducted to complement the multiplier and value chain analysis. SPA provides a more detailed description of the multiplier by tracing the transmission influence of their effects within the network of structural relations (Defourny and Thorbecke 1984).

### Multipliers

#### Output multiplier

The output multiplier is calculated considering the sum of the multiplier values of the commodities column of ${{\varvec{M}}}_{a}$. The output multiplier indicates the amount of output in all the activities of the economy needed to satisfy one unit of demand increase for the corresponding commodity (generated as a result of a unitary exogenous shock in exogenous values for the corresponding commodity).

#### Employment multiplier

The employment multiplier provides the number of jobs generated by an exogenous shock in final demand. The expression of the employment multiplier is given as: ${{\varvec{M}}}_{{\varvec{e}}}={\varvec{E}}\times {{\varvec{M}}}_{{\varvec{a}}}$, where ${\varvec{E}}$ is a diagonal matrix whose elements are the vector ${\varvec{e}}$ (containing the ratios of number of jobs per output value, in this case per million Ghanaian Cedis). The sum of the columns in the matrix ${{\varvec{M}}}_{{\varvec{e}}}$ shows the global effect on employment produced by the exogenous increase in demand. The results do not account for variables such as the quality of employment and should not be interpreted as an exact forecast of job creation due to other exogenous shocks (Philippidis et al. 2014). Nonetheless, they are useful indicators of the commodities with a greater potential to generate jobs.

#### Household income multiplier

The household income multipliers are obtained from the submatrix of ${{\varvec{M}}}_{{\varvec{a}}}$, where each value indicates the impact in each household’s income as a result of one-unit increase of final demand in the corresponding commodity. An increase in demand leads to an increase in the output and in the inputs used to produce it, including the employment generated and the income received by households. This multiplier shows the increase in household income, as well as the increase in each household group (Husain and Khondker 2016; Pal and Bandarlage 2017).

#### Value-added multiplier

The value-added multipliers represent the change in value added associated with a unit change in final demand in response to the exogenous shock (Miller and Blair 2009). Like the employment multiplier calculation, a value-added vector $\mathbf{v}$ (containing the ratios of the value added per output value of each activity) can be used to calculate the value-added multiplier. It is often argued that value added is a better measure of a sectors contribution to an economy than the output (Miller and Blair 2009).

### Value chain analysis

The value chains analysis represents the set of activities necessary to obtain a product through the different production phases up to the final consumers (Gereffi and Kaplinsky 2001; Dürr 2017). A SAM is an important tool in value chain analysis, because it allows tracing the linkages among productive activities within the economy (Trejos et al. 2004; Faße et al. 2009; Rich et al. 2011). By analysing the value chain, we can trace the impact of value added induced by the increase in demand for each commodity, embodied in the activities or group of activities in the economy. An exogenous demand shock to a sector translates into a need for an increase in production that involves an increase in the use of productive factors and inputs and consequently leads to an increase in production in other sectors. The impact of initial demand, in short, produces a cycle of effects that impacts on many sectors that can be analysed by considering value chain analysis.

The multipliers analysis and value chain analysis are complementary approaches to better understand the economic structures, the sectors interrelationships and the effect that economic shocks through policies on the most important sectors would have on the rest of the activities (Mainar-Causapé et al. 2020).

### Structural path analysis

The SPA allows analysing how multiplier effects are transmitted in the economy through other accounts, which cannot be analysed by examining the multiplier matrix alone (Roberts 2005). It is based on decomposing the multiplier effect into different paths in order to investigate the role played by the different accounts and to identify those that are important transmitters of economics influence (Defourny and Thorbecke 1984). The SPA allows to open the ‘black box’ of the SAM multipliers by understanding the structure and behavioural mechanism of the economic effects (Itoh 2016).

Effects are analysed by considering the magnitude or intensity of the ‘influence’ between the link of an account *i* with an account *j* (the direction of expenditure flows referred as ‘arc’). The SPA allows to disaggregate the influence transmitted from pole *i* (origin) to pole *j* (destination) through the analysis of the different paths. A ‘path’ includes one or more arcs connecting the account on which the exogenous shock takes place (i.e. ‘origin pole’) to the final account where income changes are evaluated (i.e. ‘destination pole’) (Defourny and Thorbecke 1984; Arndt et al. 2012).^2^ Using this technique, the effects between commodities demand and the impact generated (e.g., output, income, employment, value added) can be decomposed by considering the paths linking the accounts (Mainar-Causapé et al. 2018).

In this article, the SPA approach investigates the role played by different agriculture commodities in transmitting income to different types of households. The results of the SPA on households’ income multipliers can help policy-makers and analysts understand the channels of macroeconomic transmission between different sectors and improve the quality of policy actions.

## Results

### Output and employment multiplier analysis

The values of the output, value-added, employment and household income multipliers calculated for the Ghanaian economy in 2015 are presented in Table 5 in the appendix. Each figure of the output multiplier shows the increase in production in the economy, due to one-unit exogenous increase in demand of a commodity (i.e. increase in exports demand or government demand). Likewise, the value-added multiplier indicates the value added created by the additional production, in responses to an exogenous shock in demand. The income multipliers show the increase in each household’s group income due to a unitary injection in the exogenous demand for the commodity. Lastly, the values of the employment multiplier indicate the number of jobs generated by the exogenous increase in demand per million of additional output.

For a more in-depth analysis, Table 3 details the distribution of the output multiplier for the primary and food industry sectors. This is distributed over the rest of the economy activities with a special disaggregation of agriculture activities by region. The output multipliers show that the primary sector has a higher backward generation, with the only exception of rice and other crops. This indicates that the primary sector is crucial for the Ghanaian economy, with capacity to stimulate growth. Within agriculture, the output multiplier highlights the relevance of several products, with major values for sorghum and millet (2.68), sugar cane (2.70), tobacco (2.69), cotton and fibres (2.75) and cattle (2.70), with livestock commodities also showing large values.

**Table 3:** Distribution of the output multiplier among the economic activities, Ghana 2015. Source: author’s calculation based on Ghana SAM 2015

		Output multiplier	Agriculture in										Forestry (%)	Food industry (%)	Petroleum and mining (%)	Manuf (%)	Utilities (%)	Constr (%)	Services (%)
			Western (%)	Central (%)	Greater Accra (%)	Volta (%)	Eastern (%)	Ashanti (%)	Brong Ahafo (%)	Northern (%)	Upper East (%)	Upper West (%)							
Primary sector	Maize	2.63	1.49	3.10	1.44	6.20	9.82	4.94	11.11	7.12	3.34	1.29	0.71	5.02	4.42	2.74	4.29	0.64	32.33
	Sorghum and millet	2.68	0.96	1.07	0.49	1.69	2.39	1.35	5.31	26.58	5.99	1.38	0.70	5.44	4.62	2.64	4.39	0.74	34.24
	Rice	1.55	1.34	1.07	0.49	5.85	3.58	3.90	6.09	13.75	11.42	0.40	0.70	5.38	4.57	2.67	4.37	0.76	33.66
	Pulses	2.64	1.01	1.34	0.50	3.26	2.47	1.41	5.27	25.93	6.82	1.24	0.70	5.54	4.45	2.67	4.37	0.75	32.27
	Groundnuts	2.63	1.15	1.13	0.48	3.54	5.34	2.35	22.41	10.15	1.99	1.09	0.71	4.93	4.38	2.77	4.32	0.63	32.61
	Other oilseeds	2.57	2.15	11.75	0.61	3.20	20.73	3.76	3.19	2.39	1.41	0.19	0.70	4.80	4.55	2.74	4.24	0.56	33.03
	Cassava	2.61	6.04	4.09	2.25	4.71	8.86	5.12	4.89	4.68	1.32	0.36	0.69	4.67	5.18	2.59	4.33	0.59	39.63
	Other roots	2.64	2.06	2.16	0.50	7.30	4.82	4.55	8.13	15.04	1.49	0.34	0.70	5.06	4.73	2.64	4.33	0.65	35.52
	Vegetables	2.59	1.55	1.88	1.73	3.05	16.42	7.13	3.65	3.15	4.12	0.28	0.70	4.73	5.02	2.64	4.33	0.62	38.99
	Sugar cane	2.70	1.15	4.20	0.52	27.64	9.33	1.48	1.76	2.46	1.46	0.20	0.68	5.17	4.47	2.60	4.24	0.64	32.01
	Tobacco	2.69	1.00	1.12	0.50	1.64	2.46	1.40	1.71	37.46	1.65	0.26	0.70	5.68	4.46	2.62	4.37	0.73	32.26
	Cotton and fibres	2.75	0.92	0.99	0.49	1.55	2.32	1.32	1.69	2.74	28.59	7.93	0.71	5.55	4.49	2.80	4.46	0.92	32.53
	Fruits and nuts	2.54	1.81	6.42	4.22	2.34	8.29	2.32	8.08	3.01	1.24	0.35	0.68	4.40	5.54	2.51	4.39	0.57	43.82
	Cocoa	2.37	7.78	4.02	0.45	2.05	6.93	4.72	10.50	2.11	1.25	0.17	0.69	4.44	5.37	2.60	4.35	0.57	41.99
	Coffee and tea	2.57	0.79	0.91	0.41	2.03	2.01	1.11	20.82	1.86	1.12	0.16	0.67	4.03	6.04	2.47	4.47	0.56	50.55
	Other crops	1.20	9.78	1.09	0.60	1.70	8.10	1.33	1.61	17.81	1.67	0.32	0.69	5.05	5.03	2.61	4.35	0.64	37.61
	Cattle	2.70	1.35	1.06	3.24	7.12	2.37	3.39	1.84	13.19	13.94	0.54	0.70	5.50	4.59	2.63	4.39	0.80	33.34
	Poultry	2.50	3.14	1.27	0.66	5.17	5.52	1.61	3.53	8.99	11.60	0.54	0.69	5.07	5.13	2.59	4.42	0.73	39.35
	Other livestock	2.63	2.35	1.72	1.39	5.04	5.68	6.55	5.80	9.23	10.80	0.47	0.71	5.29	4.48	2.71	4.34	0.74	32.70
	Forestry	2.59	0.58	0.73	0.33	0.93	1.58	0.82	1.08	1.40	0.79	0.11	38.04	3.27	8.91	1.95	5.71	0.40	33.37
	Fishing	2.60	7.65	9.34	4.60	7.37	4.41	1.31	2.22	5.39	1.34	0.20	0.68	4.77	5.16	2.54	4.33	0.59	38.10
Food industry	Meat, fish and dairy	0.82	0.90	1.07	0.58	1.37	1.76	1.06	1.28	1.99	1.41	0.13	0.61	10.63	7.30	2.03	4.41	0.51	62.98
	Fruit and vegetable processing	0.89	0.70	1.53	0.68	1.06	2.65	0.98	1.71	1.50	0.80	0.13	0.58	23.07	6.55	3.13	4.34	0.46	50.14
	Fats and oils	2.60	1.26	6.01	0.42	1.91	10.72	2.13	2.02	1.76	0.98	0.14	0.54	30.55	4.25	2.02	3.59	0.43	31.27
	Grain milling	2.49	0.99%	1.31	0.61	2.84	3.93	2.19	4.58	6.54	3.02	0.56	0.51	30.31	4.43	1.95	3.56	0.47	32.20
	Sugar refining	0.64	0.64	1.52	0.35	7.55	3.33	0.86	1.12	1.50	0.87	0.12	0.59	20.02	5.90	2.22	4.11	0.56	48.75
	Other foods	1.57	1.14	1.23	0.45	1.26	2.29	1.15	1.96	1.95	0.99	0.16	0.69	23.34	6.61	1.97	4.35	0.46	50.00
	Beverages	1.56	0.61	0.86	0.34	1.11	1.84	0.88	1.38	2.36	1.00	0.17	0.75	30.83	6.05	2.91	4.13	0.44	44.35
	Tobacco processing	1.56	0.54	0.66	0.30	0.87	1.43	0.75	0.99	6.26	0.78	0.11	1.16	33.48	5.87	3.68	3.59	0.43	39.09

Rows indicate the output multiplier effect per each commodity and its distribution (in %) among the different activities

Analysing the effects of the output multiplier distributed among the different activities, for agricultural commodities around 50% of the effect is concentrated in the households as producing activities. Most agricultural products have more impact on household activities in the southern regions, however, sorghum and millet, pulses, tobacco, and cotton and fibres show more impact towards the northern regions. In the case of livestock, the impact is distributed roughly equally in both zones.

Regarding the impact concentrated in the north zone, sorghum, pulses, and tobacco predominate for the Northern region, while cotton is mostly concentrated in the Upper East and cattle is distributed in both regions. This shows that the Upper West region receives little impact from changes demand for agricultural commodities, with only a small impact from cotton.

In the southern regions, the impacts are more dispersed. However, there is a clear influence of agricultural commodities on the Volta, Eastern and Brong Ahafo regions. According to the country's geographical information, these regions have a coast on Lake Volta, which is used for irrigation, fishing, and also to generate electricity and provide inland transportation. The region that is least impacted by increasing demand for agriculture-related commodities is clearly Greater Accra, where the country's capital is located and stands out as a mainly urban region.

Many of the commodities classified within the food industry sector, such as meat, fish and dairy, fruit and vegetable processing and sugar refining have very low multiplier values both in production and value added. Within this group, only fats and oils and grain milling have higher output and value-added multiplier. The distribution of the multiplier impact is concentrated on activities related to services and food industry; however, fats and oils, and grain milling show a greater impact share towards household activities. In the case of grain milling its multiplier effects are distributed in both regions, with the Northern region and to some extent the Upper East region standing out in the north.

**Fig. 2:**
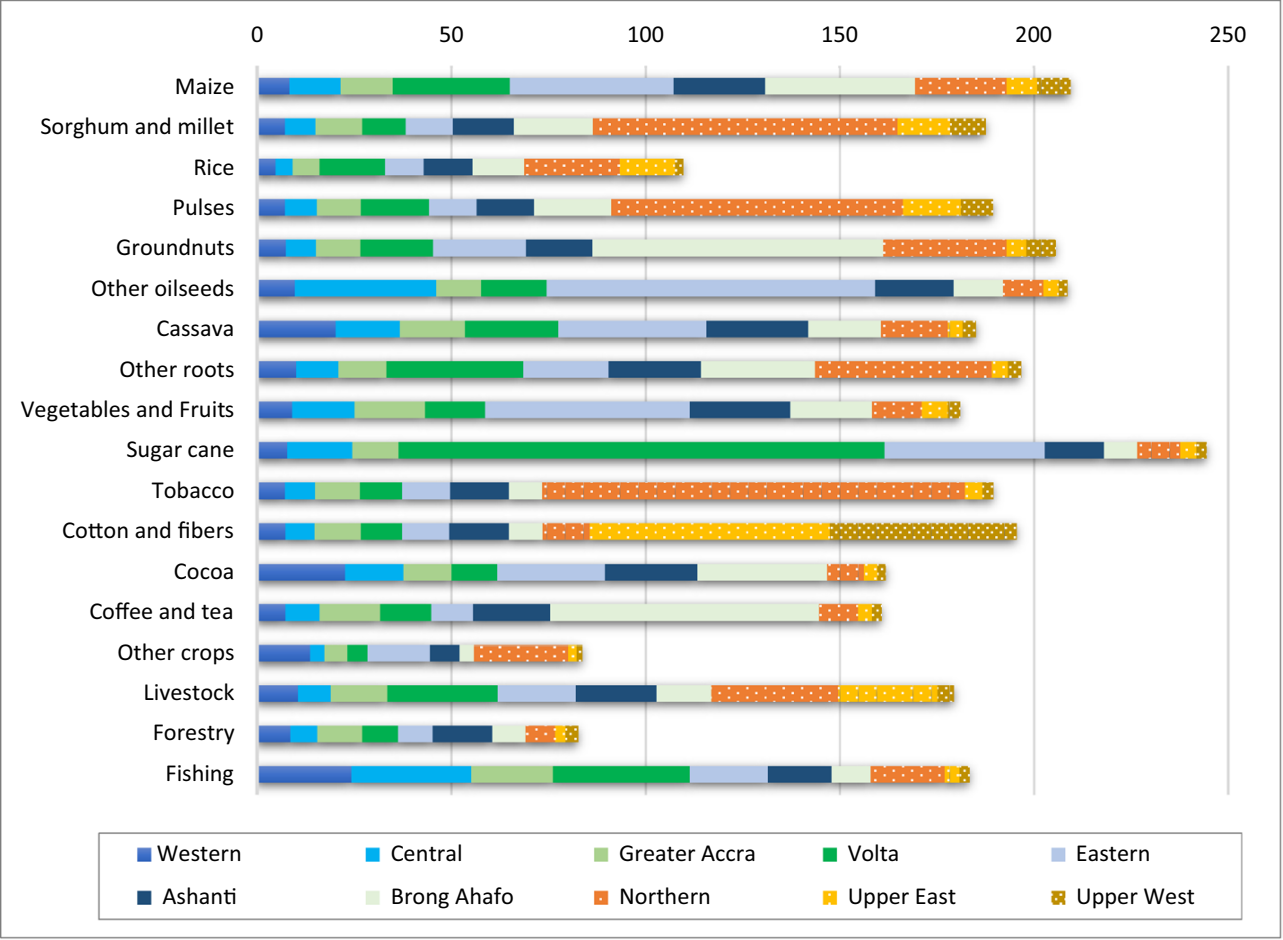
Employment multipliers for primary sector in Ghana 2015 by region. Number of people jobs generated (by region) per million Ghanaian Cedis of output value (Source: author’s calculation based on Ghana SAM 2015)

The employment multipliers show the number of jobs generated per million Ghanaian Cedis of exogenous final demand. The employment multiplier has large values for most primary products given that they are labour-intensive. The largest effects are for sugar cane, groundnuts, maize and oilseeds. Sugar cane has an employment multiplier of 244.40, meaning that 244.40 jobs will be generated in the economy per million Ghanaian Cedis of final demand. Only rice, other crops and forestry, generate below-average employment. Figure 2 shows the regional employment generated due to an increase on final demand on the primary sector commodities. For most agriculture commodities, over 70% of jobs created are concentrated in agriculture activities carried out by households. The most important of these are those in the southern regions, except for tobacco, cotton, sorghum, and pulses, where the number of jobs generated in the northern regions stands out. The north is also notable to a lesser extent for livestock, and for rice and other crops although these have a low multiplier value.

In the case of employment, it is also clear that those commodities whose impact is concentrated in activities in the north are mainly concentrated in the Northern region. However, cotton is distributed between Upper West and Upper East, and livestock is distributed between Northern and Upper East.

The analysis of the employment multiplier also provides information on the distribution of employment generated considering classifications according to education attainments. As a result, the primary sector generates over 50% of the employment to “unskilled workers”. Only for cocoa, the number of additional jobs has a similar distribution towards the group of workers classified as “semi-skilled” (47%) and “unskilled” (51%).

For the food industry, the employment multiplier shows low values because the sector is more capital intensive, and the jobs generated are concentrated among “semi-skilled” workers. Within this group, only the employment multiplier of fats and oils and grain milling stand out, with slightly above average values and whose jobs generated impact predominates for “unskilled workers” as in the primary sector. Regarding the impact on employment generated by region, commodities within the food industry have a greater impact on the southern regions, with an impact also in the northern regions for tobacco processing and cereal milling.

Comparing the employment multiplier with products that have high output effect it can be observed that within the primary sector, all the commodities that show large output multipliers also have large employment multiplier, except for forestry.

Table 5 in the appendix also shows the multipliers for other commodity groups (manufactured, petroleum and mining, utilities and construction sectors), even though some products have an above average output multiplier, they do not automatically generate high value-added multiplier and none of them stands out in terms of job creation.

For the manufacturing sector, only wood and paper have a slightly above average output multiplier, the impact of which is more concentrated in forestry, manufacturing, and services activities. The service sectors show above-average values in terms of output and value added for trade, information and communication, finance and insurance, public administration, health, and education, but significantly lower in terms of employment. Only wholesale and retail trade, accommodation and food services, education, health and other services have employment linkages above the global average. The impact of the services multiplier is clearly concentrated on services-related activities.

In terms of employment, commodities related to manufacturing and services generate impact mostly in the southern regions, in Ashanti and Greater Accra, except in the case of non-metal minerals, which is mainly in the Central region and petroleum also distributed among the Western region. Considering the employment by skill-level, for manufacturing, 60–70% of jobs created are directed towards "semi-skilled workers” and in the case of services, it also stands out but to a lesser extent, with increasing influence towards jobs "skilled workers".

### Households’ income multiplier analysis

The described SAM includes a special disaggregation for households, divided between rural and urban zones and disaggregated by regions. Additionally, the SAM as disaggregates households also considering income quintiles. This is relevant for the analysis of impacts on household’s income. This sub-section analyses how shocks in final demand for each commodity affect household income considering the different household groups by region.

The income multipliers are shown in Table 4 for primary sector commodities (details for all the commodities are presented in Table 5), illustrating the distribution between rural and urban and across regions. Most of the primary sector commodities generates more than 80% of the income through households located in the southern regions. It is only for sorghum, pulses, roots, tobacco, cotton, and livestock that incomes predominate in the northern regions. Income generation impacts from tobacco, sorghum, and pulses are mainly concentrated in households of the Northern region, cotton in households of the Upper East and Upper West, and livestock in households of the Northern and Upper East regions.

**Table 4:** Income generation effects by households’ group and region. Source: author’s calculation based on Ghana SAM 2015

	Household income multiplier	Rural (%)	Urban (%)	Southern regions							Northern regions		
				Western (%)	Central (%)	Greater Accra (%)	Volta (%)	Eastern (%)	Ashanti (%)	Brong Ahafo (%)	Northern (%)	Upper East (%)	Upper West (%)
Maize	1.61	54.3	45.7	5.7	7.9	13.6	10.2	16.8	15.7	16.1	9.1	2.8	2.1
Sorghum and millet	1.56	50.9	49.1	5.4	5.9	13.7	6.6	8.5	12.5	9.6	30.3	5.0	2.4
Rice	0.90	51.5	48.5	5.9	5.9	13.7	10.9	10.1	15.6	10.7	17.0	9.0	1.3
Pulses	1.55	51.9	48.1	5.4	6.1	13.2	8.1	8.5	12.2	9.4	29.3	5.6	2.3
Groundnuts	1.62	53.9	46.1	5.2	5.7	12.5	7.5	11.5	12.4	29.4	12.0	1.9	1.9
Other oilseeds	1.59	55.1	44.9	6.3	16.9	13.2	7.0	29.1	14.4	6.5	4.2	1.4	0.9
Cassava	1.52	50.6	49.4	10.7	9.8	17.1	9.0	16.5	18.2	9.2	7.0	1.5	1.1
Other roots	1.57	52.3	47.7	6.4	7.2	14.0	11.5	11.3	16.3	12.9	17.6	1.6	1.1
Vegetables	1.51	51.6	48.4	6.1	7.2	15.9	7.5	25.5	20.2	7.6	5.5	3.5	1.0
Sugar cane	1.65	55.4	44.6	5.5	9.3	13.8	30.5	16.5	12.3	5.0	4.6	1.6	1.0
Tobacco	1.59	51.8	48.2	5.3	5.8	13.2	6.4	8.4	12.1	5.0	41.0	1.9	1.1
Cotton and fibres	1.53	50.5	49.5	5.8	5.9	13.7	7.8	9.0	13.0	5.4	6.2	22.6	10.7
Fruits and nuts	1.44	47.0	53.0	6.5	12.8	20.4	6.7	16.1	16.0	13.7	5.4	1.5	1.0
Cocoa	1.37	49.9	50.1	12.6	9.9	15.9	6.3	14.2	18.1	16.5	4.3	1.4	0.8
Coffee and tea	1.40	44.1	55.9	5.6	7.0	18.5	6.5	8.4	15.9	31.6	4.3	1.4	0.8
Other crops	0.70	50.9	49.1	14.6	6.3	15.1	6.2	15.5	13.3	5.1	21.0	1.8	1.1
Cattle	1.55	50.1	49.9	6.0	6.0	16.6	12.5	8.8	15.3	5.4	16.7	11.0	1.6
Poultry	1.39	48.6	51.4	8.1	6.7	15.9	10.5	12.9	14.5	7.9	12.5	9.5	1.5
Other livestock	1.56	52.5	47.5	6.8	6.6	14.1	9.9	12.5	18.3	10.1	12.0	8.4	1.4
Forestry	1.21	30.9	69.1	7.5	9.7	28.9	7.3	10.8	21.8	7.7	4.3	1.3	0.7
Fishing	1.52	49.4	50.6	12.4	15.5	19.6	11.6	11.1	13.7	5.9	7.8	1.5	0.9

Rows indicate the household income multiplier per commodity and its composition (%) considering the distribution within rural and urban areas and also the shares for each region

The income generated through the promotion of these agriculture commodities are concentrated in the rural zones, which benefits the income generation of rural households. The impact on rural areas is particularly strong in the northern regions. Furthermore, the results of the analysis show that considering the average impact on each quintile group, the income received by 60% of the population with the lowest income (classified among quintile one, two and three) comes from the primary and food sector. Hence, this multiplier shows the importance of the primary sector in household income generation, especially for low-income rural households in the northern regions.

### Value chain and structural path analysis

**Fig. 3:**
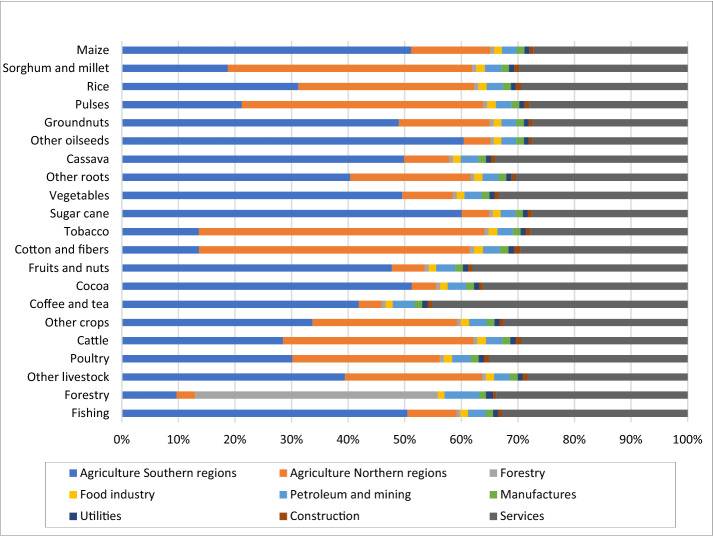
Distribution across each activity of the value added generated due to an exogenous impact on primary commodities demand (Source: own elaboration)

The value chain analysis traces the impact of value added induced by the increase on each commodity demand, embodied on the activities or group of activities in the economy. Considering the primary sector, Fig. 3 shows how the value added generated by an exogenous increase in demand for each commodity is distributed across each group of activities. The analysis of the primary sector shows that the value added generated is mainly concentrated in agriculture activities, highlighting the impact in the southern regions for most commodities. In line with the output multiplier results, the northern regions have large impacts on the value added when it comes to sorghum, pulses, tobacco, and cotton and fibres.

**Fig. 4:**
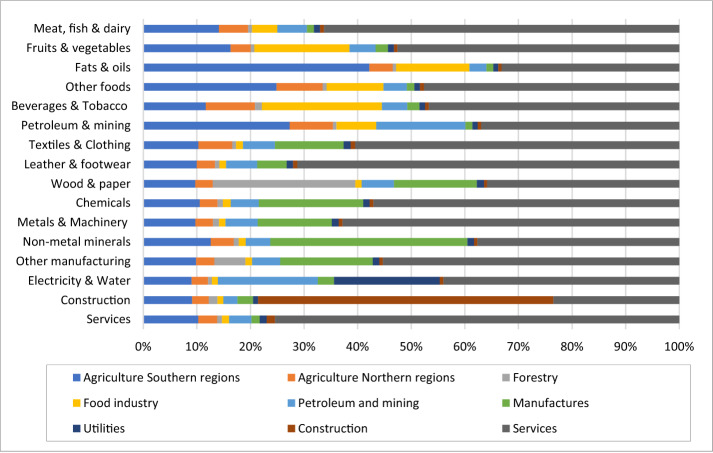
Distribution across each activity of the value added generated due to an exogenous impact on others commodities demand (Source: own elaboration)

The distribution of the value added generated by other activities is shown in Fig. 4. The value added generated is mainly directed towards services and towards the sector to which the commodity belongs. Mainly for food industry there is still a significant impact on the agricultural sector for both the southern and northern regions.

Table 4 shows the household income multiplier and its distribution by rural/urban areas or by regions, however, it does not elaborate on each channel's impact or on the breakdown of each region within rural areas. Using the SPA methodology, it is possible to decompose the household income multiplier to evaluate the impact channels through which income flows to rural households in the north. The analysis focuses on agricultural commodities, especially those that are most influential in the northern regions. In this way, it is possible to decompose their income multiplier effect by showing the paths through which agricultural commodities influence the incomes of rural households.

**Fig. 5:**
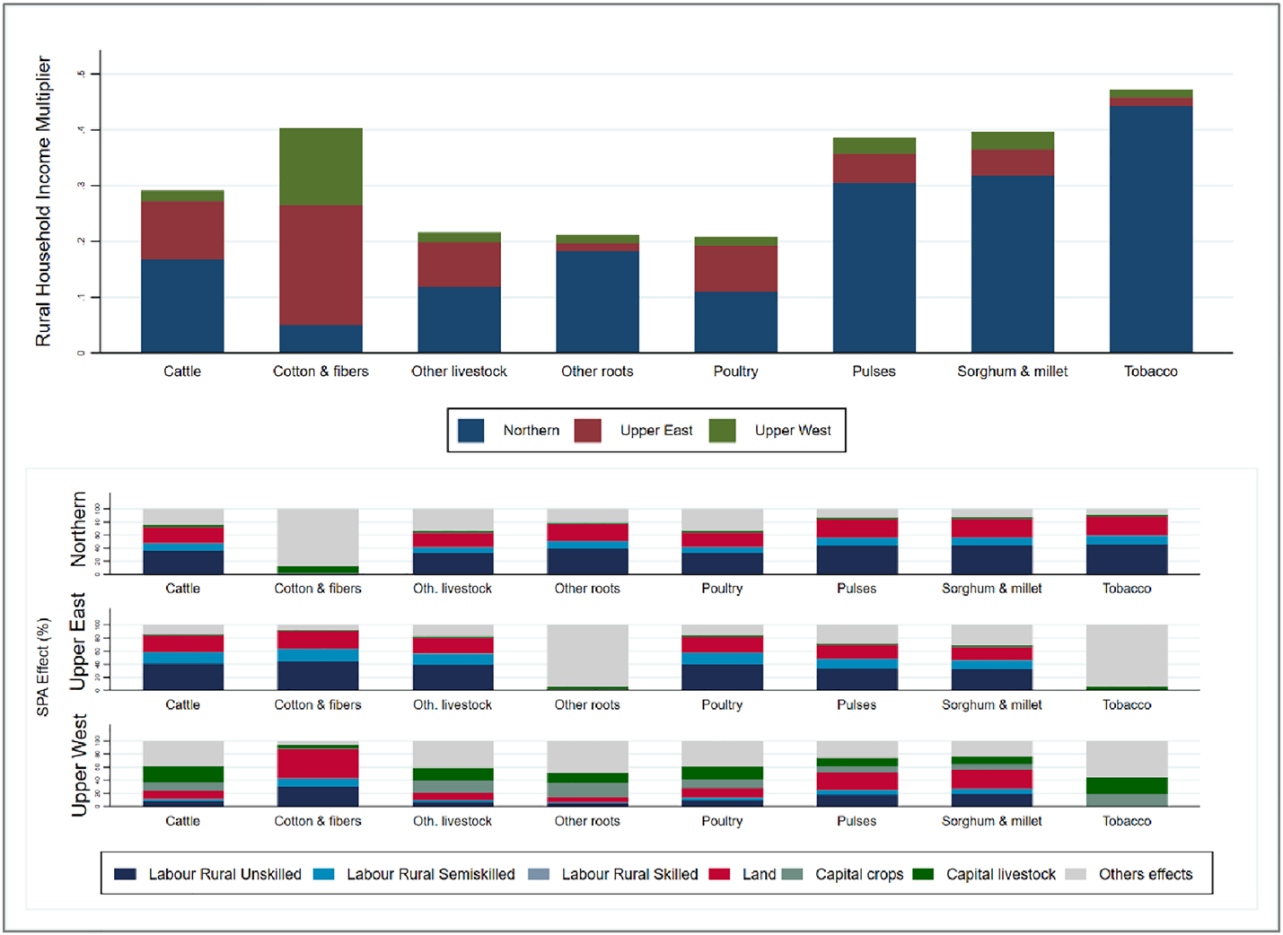
Top graph: distribution of the rural household income multiplier in the north by each region. Bottom graph: structural path analysis from agricultural commodities to rural households income by the regions in the north (Source: own elaboration)

First, for the commodities with the greatest influence of the rural income multiplier towards the northern regions, Fig. 5 (top graph) shows how the value of the multiplier is distributed within the three northern regions, to know the influence of each commodity between the regions, e.g., cotton and fibres, highlighted for the Upper East. Then, Fig. 5 (bottom graph) presents the effects resulting from the structural path analysis of the most important commodities to promote the northern regions, including the most important paths and covering a high percentage of the global influence (multiplier value). These effects are disaggregated by the three northern regions (Upper East, Upper West and Northern). Table 6 in the Appendix presents the results of the four most significant paths of economic influence between the pole of origin (commodity) and the pole of destination (rural household income in the north).

Regarding the transmission mechanisms, most of the contribution of the multiplier has an initial effect on households as producers, which generates household income though the remuneration to land use, rural unskilled and semi-skilled labour and capital (crops and livestock) (Agriculture commodity → Households as Activities → Factors of Production → Rural North Household Income). These transmission mechanisms are the most important for agricultural commodities, covering between 72 and 86% of the global multiplier effect.

In the case of ‘sorghum and millet’ at least 68.3% of the increase in rural household incomes in the northern regions due to an exogenous shock is channelled directly through an increase of production in household activities, impacting on the necessity of land (27.6%) and unskilled rural labour (40.7%) (Table 6). Furthermore, looking at Fig. 5 (top graph) it is possible to analyse how these effects are distributed for each commodity, by considering the rural income multiplier impact divided by each northern region.

## Discussion

The literature shows the importance of the agricultural sector in Ghana, not only in terms of percentage of GDP, but particularly in terms of the high percentage of the employed population, thus representing an important source of income for many households in the country.

The Food and Agriculture Sector Plan for Ghana focuses on the transformation and modernisation of the agriculture sector to achieve food security and create jobs. The main problems facing the agricultural sector in Ghana are low productivity, droughts, climate conditions and lack of infrastructure (Danso-Abbeam et al. 2019).

The cocoa production it is one of the country's most important activities, as it is the second largest producer and exporter in the world. Production is concentrated in the south, mainly in the Brong Ahafo, Western and Eastern regions, and it is mainly for export. The relevance of this sector appears in the multiplier analysis. The cocoa sector can generate economic growth, household income and employment in Ghana, but these effects are concentrated in the southern regions. As the importance of the cocoa sector in the country is already well known, this article discusses the potential of other products in the primary sector.

Agriculture is the main activity and represents the main source of income and employment for the population in the northern regions where the production potential remains largely unexploited. The characteristics that constrain the development of the agriculture in the north are related to the poor state of the infrastructures and the effects of climate change, with erratic rains including annual flooding and long dry season (Mekonnen et al. 2019). Although the Northern Savannah Ecological Zone (NSEZ) has many large, medium, and small-sized dams for multiple uses including irrigation, infrastructure are still underdeveloped (Akpoti et al. 2022). Hence, water insecurity in the northern regions during the long dry season negatively affects production, household incomes and living standards (World Bank 2017; Mekonnen et al. 2019).

Despite the inequality, the northern regions have the potential to foster agriculture and promote its development (Bawa 2019). Countries with similar conditions (e.g., Brazil) already demonstrated successful ways of overcoming such constraints focusing on agricultural transformation (World Bank 2017). The Northern Savannah Ecological Zone covers large proportion of the country with hectares of arable land and availability of water resources, which makes it suitable for agriculture production (World Bank 2017). To this end, the promotion of the region's agriculture is one of the key pillars.

The multiplier results show those commodities that, given the impact of exogenous policy on variations in demand, can generate output, value added, employment, and household income above the economy’s average. The multiplier analysis based on the SAM Ghana 2015 indicates that the primary sector commodities are crucial to Ghana's economy, with a large capacity to stimulate economic growth and employment. Hence, according to these results, promoting agriculture can generate increases in output, generating jobs and improving household income with a positive effect on poverty reduction. Moreover, these achievements can be focused on the Northern, Upper West, and Upper East regions. The key products to achieve these improvements are tobacco, cotton and fibres, sorghum and millet, pulses, other roots, livestock products and, to a lesser extent, within the food industry group, cereal milling. Furthermore, thanks to the HPHC approach included in this SAM, multiplier effects can be seen distributed with significant effect across households as producing activities by regions.

Based on the value-added multiplier results, the primary sector also stands out as one of the key sectors for promoting development in Ghana. Most of the commodities under the food industry and manufactured sectors have very low multipliers values. Despite that some commodities under services, utilities, and construction have high output multipliers, they do not automatically generate high value added or employment multipliers.

The results highlight those commodities with the greatest influence on economic growth in response to demand changes, but also those, whose impacts influence on different regions in the country. Therefore, it is recommendable to invest in those commodities that not only have a greater influence on the economic growth, but also focus on the development of the northern regions, in order to reduce inequality.

Rice is a special case. Although its multipliers values are lower than average, it shows a significant impact on the northern regions in terms of production, employment, and income generation, especially in the Northern and Upper East regions. Due to the high Ghanaian import dependency on rice, the Planting for Food and Jobs (PFJ) strategy (MoFA 2017) promotes its domestic production, however, as household consumption has also increased, production remains insufficient and still depends on rice imports (Pauw 2021). For this reason, in addition to the commodities mentioned above, the promotion of rice production would also benefit those regions in the north of the country.

## Conclusions and policy recommendations

To implement policies aimed at the country's socio-economic development, it is necessary to develop suitable databases that can be used for economic modelling. This article presents an analysis of the multipliers based on the Ghana's SAM for 2015. The significance of this new matrix is the incorporation of the HPHC approach and the high disaggregation of its accounts. This breakdown includes the disaggregation of several products within the primary sector and details by region, educational attainments, and rural and urban zones. These details allow for an in-depth analysis of the structure of the country's economy that enables the formulation of policies focused on equality and development in the low-income regions.

To evaluate a public policy, it is important to estimate not only socio-economic impacts, but also where the impacts will be felt (Almazán-Gómez et al. 2019). This paper analyses at the regional level the capacity of each commodity to generate output, value-added, jobs, and households’ income. In other words, multisectoral and multiregional linear models have been applied to the SAM to provide information essential for the development of socio-economic policies focused on the promotion of specific sectors and regions with most potential in Ghana. However, it should be noted that this study does not analyse environmental impacts (Tahiru et al. 2020), which should be taken into account to promote more comprehensive policy recommendations.

This paper shows how many agriculture commodities are suitable to be promoted through policies since they will have large backward impact on the rest of the economy output and value added. Moreover, these products also have a higher employment and household income multiplier, showing that changes in final demand can also translate into job and income creation. Cotton and fibres, sugar cane, sorghum and millet, maize, livestock, tobacco, roots, pulses, and groundnuts have the highest multiplier values. From a policy point of view, these products have the potential to promote economic growth and employment in the rest of the economy sectors and will be suitable for promoting through exogenous policies. Special attention should be paid to those commodities that have also the potential to promote the northern regions (such as sorghum and millet, pulses, tobacco, cotton and fibres and livestock).

The SPA shows how the influence on rural household income is transmitted in each northern region, demonstrating that income boosting in the northern areas of Ghana requires taking into account regional specificities. For example, an increased demand of pulses, sorghum, and millet has a similar influence in the Northern and the Upper East regions, highlighting the effects derived by labour rural unskilled, followed by land and labour rural semi-skilled. However, the same commodities generate higher income on rural households in the Upper West region transmitted by a larger influence of land, labour rural unskilled and livestock and crops capital.

Overall, the SPA shows that when boosting demand for the specified commodities in Upper East and Northern, income effects are more related to rural labour (unskilled and semi-skilled) and land. However, for the Upper West the effects are more diversified across income streams, including mainly unskilled rural labour, land, capital crops and capital livestock, which are not prominent in the other regions. Finally, for those commodities where the region captures the smallest share of the multiplier effect, the influence is more associated with ‘other effects’, meaning that specific policies to boost demand for these commodities would produce effects that are more difficult to trace.

In summary, policies should focus on promoting Ghana's primary sector because it can improve the economy by generating more output, but also by creating jobs in many regions and consequently increasing household income. Thus, this article suggests adjusting policies and incentives in a way that promotes commercial agriculture and its better integration into markets. This involves supporting the development of exportable agricultural products and strengthening agricultural marketing and trade infrastructure and facilitation while refocusing the regional distribution of agricultural spending towards northern regions for crops with high agricultural potential.

In addition, public investment should focus on agricultural R&D to improve the productivity of the country's most important commodities, as well as related infrastructure. This will help to promote the development of the agricultural potential of the Northern Savannah Ecological Zone, with a potential positive impact on economic growth, job creation with rural income generation and poverty reduction. A special case is the Upper West, for which the impact of changes in demand for agricultural products is low, with only cotton standing out. Given that the region has a long dry season, investment in better infrastructure can ensure market access and thus the integration of this region.

The methodology is a useful tool which allows for an analysis of an economy structure and ex ante policy assessments. Nevertheless, considering the limitations of the methodology above-mentioned in the article, each multiplier value should not be taken as an exact forecast of the impact in the economy, but as an indicator of those commodities with greater impact due to demand shocks (Round 2003b; Miller and Blair 2009; Mainar-Causapé et al. 2020). Notably, the values of the employment multiplier should not be interpreted as a forecast of job creation due to exogenous shocks and should be aware that they do not consider social variables such as job quality. Nevertheless, they are useful as an indicator of the accounts of the economy with the highest employment generation potential (Philippidis et al. 2014).

## Data Availability

The data that support the findings of this study can be obtained from the authors upon request.

## Appendix

See Tables 5, 6.

**Table 5:** Linear multipliers for Ghana 2015. Source: own work

Sector	Description	Multipliers				Sector	Description	Multipliers			
		Output	Value added	Household Income	Employment			Output	Value added	Household income	Employment
Primary sector	Maize	2.63	1.7	1.61	209.36	Petroleum and mining	Crude oil	1.86	1.03	0.74	83.25
	Sorghum and millet	2.68	1.65	1.56	187.58		Other mining	2.28	0.68	0.57	48.41
	Rice	1.55	0.95	0.90	109.73		Petroleum	2.07	0.87	0.70	61.33
	Pulses	2.64	1.64	1.55	189.43	Manufactured	Textiles	1.49	0.71	0.63	75.55
	Groundnuts	2.63	1.7	1.62	205.53		Clothing	1.6	0.77	0.68	64.03
	Other oilseeds	2.57	1.68	1.59	208.6		Leather and footwear	0.78	0.38	0.33	32.14
	Cassava	2.61	1.62	1.52	185.07		Wood and paper	2.37	1	0.87	71.03
	Other roots	2.64	1.66	1.57	196.63		Chemicals	0.81	0.37	0.33	28.6
	Vegetables	2.59	1.61	1.51	189.69		Non-metal minerals	0.91	0.43	0.40	54.3
	Sugar cane	2.7	1.75	1.65	244.4		Metals and metal	0.65	0.3	0.27	22.43
	Tobacco	2.69	1.68	1.59	189.44		Machinery and equipment	0.23	0.11	0.10	9.45
	Cotton and fibres	2.75	1.61	1.53	195.49		Other manufacturing	0.77	0.37	0.33	35.9
	Cocoa	2.37	1.46	1.37	161.83	Utilities	Electricity, gas and steam	2.61	0.89	0.72	62.39
	Coffee and tea	2.57	1.5	1.40	160.72		Water supply and sewage	2.28	0.77	0.66	47.9
	Other crops	1.2	0.74	0.70	83.69	Construction	Construction	2.21	1.34	1.16	67.09
	Cattle	2.7	1.64	1.55	186.07	Services	Wholesale and retail trade	2.73	1.28	1.15	116.2
	Poultry	2.5	1.49	1.39	167.99		Transportation and storage	2.15	1.19	1.04	90.94
	Other livestock	2.63	1.64	1.56	189.07		Accommodation and food services	2.16	1.24	1.11	140.1
	Forestry	2.59	1.39	1.21	82.62		Information and communication	2.72	1.22	1.07	75
	Fishing	2.6	1.63	1.52	183.35		Finance and insurance	2.56	1.41	1.26	86.77
Food industry	Meat, fish and dairy	0.82	0.4	0.36	35.71		Real estate activities	2.37	1.09	0.94	52.56
	Fruit and vegetable processing	0.89	0.44	0.39	46.12		Business services	1.4	0.56	0.49	40.13
	Fats and oils	2.6	1.32	1.21	139.63		Public administration	2.49	1.2	1.08	90.67
	Grain milling	2.49	1.14	1.06	133.56		Education	2.8	1.47	1.37	155.62
	Sugar refining	0.64	0.31	0.28	33		Health and social work	2.56	1.18	1.09	120.96
	Other foods	1.57	0.76	0.68	72.22		Other services	2.2	1.43	1.29	121.55
	Beverages	1.56	0.75	0.67	74.84						
	Tobacco processing	1.56	0.69	0.62	59.59						

Employment: jobs created per million Ghanaian Cedis

**Table 6:** Structural path analysis from agricultural commodities to north rural households income. Source: own elaboration

Origin: path 1 → path 2 → path 3 → destination: path 4	Influence in the multiplier (%)	Cumulative share (%)
Sorghum and millet → households as activities → labour-rural-unskilled → rural HH income (north)	40.7	40.7
Sorghum and millet → households as activities → labour-rural-semiskilled → rural HH income (north)	12.8	53.5
Sorghum and millet → households as activities → land → rural HH income (north)	27.6	81.1
Sorghum and millet → households as activities → capital-crops → rural HH income (north)	1.0	82.1
Sorghum and millet → households as activities → capital-livestock → rural HH income (north)	1.8	**83.9**
Pulses → households as activities → labour-rural-unskilled → rural HH income (north)	40.5	40.5
Pulses → households as activities → labour-rural-semiskilled → rural HH income (north)	12.8	53.3
Pulses → households as activities → land → rural HH income (north)	27.3	80.6
Pulses → households as activities → capital-crops → rural HH income (north)	1.1	81.7
Pulses → households as activities → capital-livestock → rural HH income (north)	1.9	**83.6**
Other roots → Households as activities → labour-rural-unskilled → rural HH Income (North)	34.7	34.7
Other roots → households as activities → labour-rural-semiskilled → rural HH income (north)	10.3	45.0
Other roots → households as activities → land → rural HH income (north)	22.8	67.8
Other roots → households as activities → capital-crops → rural HH income (north)	2.2	70.0
Other roots → households as activities → capital-livestock → rural HH income (north)	2.0	**72.0**
Tobacco → households as activities → labour-rural-unskilled → rural HH income (north)	43.1	43.1
Tobacco → households as activities → labour-rural-semiskilled → rural HH income (north)	12.8	55.8
Tobacco → households as activities → land → rural HH income (north)	12.8	83.9
Tobacco → households as activities → capital-crops → rural HH income (north)	0.8	84.7
Tobacco → households as activities → capital-livestock → rural HH income (north)	1.5	**86.2**
Cotton and fibres → households as activities → labour-rural-unskilled → rural HH income (north)	34	34.0
Cotton and fibres → households as activities → labour-rural-semiskilled → rural HH income (north)	14.9	48.9
Cotton and fibres → households as activities → land → rural HH income (north)	29.8	78.7
Cotton and fibres → households as activities → capital-crops → rural HH income (north)	0.9	79.6
Cotton and fibres → households as activities → capital-livestock → rural HH income (north)	3.3	**82.9**
Cattle → households as activities → labour-rural-unskilled → rural HH income (north)	36.6	36.6
Cattle → households as activities → labour-rural-semiskilled → rural HH income (north)	13	49.6
Cattle → households as activities → land → rural HH income (north)	23.8	73.4
Cattle → households as activities → capital-crops → rural HH income (north)	1.4	74.7
Cattle → households as activities → capital-livestock → rural HH income (north)	3.1	**77.9**
Poultry → households as activities → labour-rural-unskilled → rural HH income (north)	33.8	33.8
Poultry → households as activities → labour-rural-semiskilled → rural HH income (north)	12.3	46.0
Poultry → households as activities → land → rural HH income (north)	22.1	68.2
Poultry → households as activities → capital-crops → rural HH income (north)	1.6	69.8
Poultry → households as activities → capital-livestock → rural HH income (north)	3.1	**72.9**
Other livestock → households as activities → labour-rural-unskilled → rural HH income (north)	32.9	32.9
Other livestock → households as activities → labour-rural-semiskilled → rural HH income (north)	11.8	44.8
Other livestock → households as activities → land → rural HH income (north)	21.5	66.2
Other livestock → households as activities → capital-crops → rural HH income (north)	2.2	68.4
Other livestock → households as activities → capital-livestock → rural HH income (north)	3.2	**71.6**

Starting with exogenous changes in the commodities (Pole 1), represents the pathways of the structural relationships (Pole 2 and Pole 3) with the greatest influence on the income of rural household in the north (Pole 4). Bold values indicate the total cumulative share (%) for each group

## Abbreviations

CGE: Computable general equilibrium models
FASDEP: Food and Agriculture Sector Development Policy
GLSS7: Ghana Living Standard Survey Round 7
GSS: Ghana Statistical Services
GSSP: Ghana Strategy Support Programme
HPHC: Home production for home consumption
IFPRI: International Food Policy Research Institute
ISSER: Institute for Statistical, Social and Economic Research
METASIP: Medium-Term Agriculture Sector Investment Plan
NSEZ: Northern Savannah Ecological Zone
PFJ: Planting for food and jobs
SAM: Social Accounting Matrices
SPA: Structural path analysis

## Footnotes

1 When referring to the northern regions, it includes the regions known as: Northern, Upper West and Upper East. When Northern is write with capital letter, it refers specifically to one of this northern regions (Northern).

2 For more details on the calculations, see Defourny and Thorbecke (1984).
